# Cardiac imaging after ischemic stroke: Echocardiography, CT, or MRI?

**DOI:** 10.1007/s00059-019-4803-x

**Published:** 2019-04-02

**Authors:** S. Camen, K. G. Haeusler, R. B. Schnabel

**Affiliations:** 1 Department of General and Interventional Cardiology, Building O70, University Heart Center Hamburg, Martinistraße 52, 20246 Hamburg, Germany; 2 DZHK (German Center for Cardiovascular Research), partner site Hamburg/Kiel/Luebeck, Hamburg, Germany; 3 Department of Neurology, Universitätsklinikum Würzburg, Würzburg, Germany

**Keywords:** Brain ischemia, Computed tomography, X‑ray, Embolism, Magnetic resonance imaging, Echocardiography, transesophageal, Hirnischämie, Computertomographie, Embolie, Magnetresonanztomographie, Echokardiographie, transösophageale

## Abstract

About 20–25% of all ischemic strokes are of cardioembolic etiology, with atrial fibrillation and heart failure as the most common underlying pathologies. Diagnostic work-up by noninvasive cardiac imaging is essential since it may lead to changes in therapy, e.g., in—but not exclusively—secondary stroke prevention. Echocardiography remains the cornerstone of cardiac imaging after ischemic stroke, with the combination of transthoracic and transesophageal echocardiography as gold standard thanks to their high sensitivity for many common pathologies. Transesophageal echocardiography should be considered as the initial diagnostic tool when a cardioembolic source of stroke is suspected. However, to date, there is no proven benefit of transesophageal echocardiography-related therapy changes on the main outcomes after ischemic stroke. Based on the currently available data, cardiac computed tomography and magnetic resonance imaging should be regarded as complementary methods to echocardiography, providing additional information in specific situations; however, they cannot be recommended as first-line modalities.

## Zusammenfassung

Etwa 20–25 % der ischämischen Schlaganfälle sind kardioembolischer Genese mit Vorhofflimmern und Herzinsuffizienz als den häufigsten Ursachen. Die diagnostische Aufarbeitung mittels kardialer Bildgebung ist unerlässlich, da sie zu therapeutischen Veränderungen führen kann, beispielsweise, aber nicht ausschließlich, hinsichtlich der sekundären Schlaganfallprävention. Die Echokardiographie bleibt die Basis der kardialen Bildgebung nach einem ischämischen Schlaganfall mit der Kombination aus transthorakaler und transösophagealer Echokardiographie als Goldstandard aufgrund der hohen Sensitivität für die häufigsten pathologischen Veränderungen. Bei einem klinischen Verdacht auf einen kardioembolischen Schlaganfall sollte die transösophageale Echokardiographie als initiale Untersuchung erwogen werden. Allerdings wurde bisher nicht anhand einer Studie nachgewiesen, dass die aufgrund der transösophagealen Echokardiographie geänderten therapeutischen Maßnahmen für die Hauptendpunkte nach Schlaganfall von Vorteil seien. Auf der Grundlage der derzeit verfügbaren Studien sollten die kardiale Computertomographie und die kardiale Magnetresonanztomographie als ergänzende Methoden zur Echokardiographie betrachtet werden, die in bestimmten Situationen zusätzliche Informationen liefern können, aber nicht als Standarddiagnostik dienen sollten.

In Europe, about 87% of all strokes are considered to be ischemic and 13% are hemorrhagic strokes [1]. According to the TOAST (Trial of ORG 10172 in Acute Ischemic Stroke) criteria [2], ischemic stroke can be divided into five categories based on the assumed etiology:

- Large-artery atherosclerosis,
- Cardioembolism,
- Small-vessel occlusion,
- Stroke of other determined etiology,
- Stroke of undetermined (cryptogenic) etiology.

Cardioembolism accounts for about 20–25% of all ischemic strokes [3]. However, the pathogenesis of stroke remains unexplained in up to 30% of all patients (so-called cryptogenic stroke). This may be due to competing causes of stroke (i.e., ipsilateral carotid stenosis and atrial fibrillation [AF]), insufficient diagnostic work-up, or standard diagnostic work-up without relevant pathological findings. Based on long-term electrocardiogram (ECG) monitoring, it is assumed that a significant proportion of suspected cryptogenic strokes might have been of cardioembolic origin [4–6]. Compared with non-cardioembolic strokes, cardioembolic strokes are more severe and are associated with increased mortality and disability and a high recurrence rate [7, 8]. A thorough work-up after stroke is important since it might lead to changes in secondary stroke prevention [9].

For cardiac sources of embolism, a distinction was made between high-risk and low-risk cardiovascular sources (Table 1). The most common causes of cardioembolism are AF, left ventricular (LV) dysfunction and LV thrombus, endocarditis, prosthetic valves, and patent foramen ovale (PFO), with AF accounting for the majority of cases [10]. Although screening for AF by ECG, monitoring during stroke unit stay, and, if suspicion for AF is high, additional longer-term ECG monitoring have become routine, sequential cardiac imaging after ischemic stroke is less well established [11]. This is usually achieved with transthoracic (TTE) or transesophageal echocardiography (TEE). Transthoracic echocardiography has been increasingly performed as part of stroke work-up in the past few years, whereas TEE is used infrequently [12–15]. In recent years, some studies have assessed the potential benefits of cardiac computed tomography (CT) and magnetic resonance imaging (MRI) regarding the evaluation of sources of cardioembolism.

**Table 1:** Potential sources of (cardio-)embolic stroke according to TOAST criteria (modified according to [2, 19])

High risk of embolism	Intracardiac thrombi	Left atrium/left atrial appendage (LAA)	Atrial fibrillation	
			Atrial flutter	
			LAA thrombus during sinus rhythm	
		Left ventricle (LV)	Ischemic cardiomyopathy (ICM)	Acute myocardial infarction (<4 weeks)
				Chronic ICM, especially in cases of LV aneurysm
			Dilated cardiomyopathy	
			Other cardiomyopathies	
		Prosthetic valves and devices		
	Endocarditis	Native valves		
		Prosthetic valves		
	Intracardiac tumors	Myxoma		
		Papillary fibroelastoma		
		Other tumors		
	Aortic atheroma			
Low/uncertain risk of embolism	Mitral valve prolapse			
	Calcification of mitral/aortic valve			
	Spontaneous echocardiographic contrast			
	Interatrial septum anomalies	Patent foramen ovale		
		Atrial septum aneurysm		
		Atrial septum defect		

*TOAST* Trial of ORG 10172 in Acute Ischemic Stroke

**Fig. 1:**
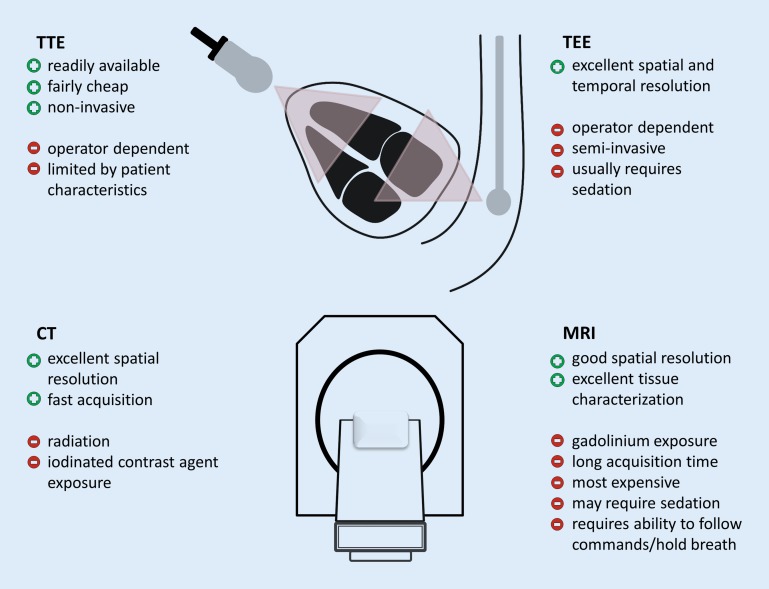
Advantages (+) and disadvantages (−) of different cardiac imaging methods. General benefits and limitations of transthoracic echocardiography (*TTE*), transesophageal echocardiography (*TEE*), cardiac computed tomography (*CT*), and cardiac magnetic resonance imaging (*MRI*) are shown

The objective of this work is to provide an overview of the available clinical data on cardiac imaging after acute ischemic stroke including cardiac CT and MRI. Imaging results may vary significantly depending on the settings, machines, and protocols used for imaging, but details on technical aspects are beyond the scope of this review. The general advantages and disadvantages of all four imaging modalities are summarized in Fig. 1.

## Cardiac imaging after acute ischemic stroke

### Transthoracic vs. transesophageal echocardiography

Both TTE and TEE have specific advantages and disadvantages. Transthoracic echocardiography is well suited to imaging of anterior cardiac structures including the ventricles, while posterior structures such as the left atrium (LA) and the left atrial appendage (LAA) are less well captured because of the greater distance to the probe. Image quality may be limited owing to specific patient characteristics (e.g., chest wall abnormalities, lung disease, or obesity). On the other hand, TEE has hardly any acoustic limitations thanks to the proximity of the imaging probe to the LA and thoracic aorta. Therefore, it is intended for diagnosis of abnormalities not detected on TTE (Table 2). Despite its semi-invasive nature, TEE is considered to be a safe procedure and serious complications such as gastroesophageal injuries, major bleeding, or sustained dysrhythmias are rare [16]. Nevertheless, it is often necessary to use mild sedation and certain conditions constitute a contraindication for TEE (Table 3).

**Table 2:** Comparison of cardiac imaging methods in the evaluation of cardioembolism etiology

	TTE	TEE	Cardiac CT	Cardiac MRI
LV thrombus	+ (++^b^)	+(++^b^)	++	+++^a^
Cardiomyopathy	++	+	+	+++^a^
LA/LAA	+	+++^a^	+++	++
Patent foramen ovale	++^c^	+++^c a^	+	+
Valvular disease	++	+++^a^	++(+++^d^)	+
Intracardiac tumors	+	++	+++	+++^a^
Aortic atheroma	−	++	+++ (Angiography)^a^	++ (Angiography)

*CT* computed tomography, *LA* left atrium, *LAA* left atrial appendage, *LV* left ventricular, *MRI* magnetic resonance imaging, *TEE* transesophageal echocardiography, *TTE* transthoracic echocardiography

^a^Diagnostic gold standard with regard to each pathology

^b^Use of echocardiographic contrast agent

^c^Including the so-called bubble test (intravenous application of agitated air–saline solution)

^d^Superior to TEE with regard to para-/perivalvular extent of the disease

**Table 3:** Contraindications to TEE examination [16, 92]

Absolute	Relative
*Perforated viscus* *Esophageal pathologies* Tumor Perforation Large diverticulum Obstruction, stricture *Upper gastrointestinal bleeding* *Recent upper gastrointestinal surgery*	*Esophageal varices* *Coagulation disorders* Severe thrombocytopenia (<50,000/µl) Elevated international normalized ratio (>4) Prolonged partial thromboplastin time (>150 s) *History of dysphagia* *Recent upper gastrointestinal bleeding* *Esophagitis, peptic ulcer disease* *Impaired neck mobility, radiation of neck and mediastinum*

Current guidelines do not provide clear recommendations regarding the use of echocardiography for stroke patients. Whereas the guideline on acute stroke management issued by the American Heart Association [17] does not mention echocardiography at all, the European Stroke Organization guideline [18] recommends echocardiography in selected patients (Class III, Level B recommendation). However, the European Stroke Organization guideline does not provide any recommendation as to the choice of TTE or TEE. Current echocardiography guidelines recommend routine use of TTE as a screening tool for potential cardiac sources of embolism [19, 20]. Transesophageal echocardiography might also be considered as an initial or supplemental test in specific patients, e.g., in cases of suspected endocarditis if TTE is normal. Transesophageal echocardiography is not recommended if potential results will not change the therapeutic decisions. Owing to the lack of clear recommendations, the diagnostic use of TEE and TTE varies considerably between stroke centers [21].

In a predominantly prospective, two-center study of 824 ischemic stroke patients, 95% of all patients with cardiac pathologies identified by TEE had an abnormal TTE and/or AF [22]. Furthermore, TEE did not result in additional findings that changed the therapeutic regimen to oral anticoagulation (OAC) in patients with normal TTE and sinus rhythm. In another prospective nonrandomized single-center study of 231 patients with ischemic stroke or transient ischemic attack (TIA) and no indication for OAC after routine work-up (not including prolonged rhythm monitoring), de Bruijn et al. [23] found a thrombus in 18% of the patients undergoing TEE compared with 2% of patients undergoing TTE. Almost all of these thrombi were located in the LAA. Harloff et al. [24] evaluated the additional benefit of TEE compared with routine work-up in a prospective, single-center study of 503 ischemic stroke patients. The TEE findings led to OAC administration in 8% of 212 patients with as-yet cryptogenic ischemic stroke. However, there was only a single case with an evidence-based indication for OAC (LA thrombus), while in all other cases the indication for OAC was debatable (e.g., LA spontaneous echo contrast [SEC] or aortic thrombi). In a retrospective analysis of 441 unselected patients with ischemic stroke or TIA, TEE was superior to TTE in identifying potential sources of cardioembolism [25]. The difference was mainly due to detection of LAA thrombi, aortic thrombi, and PFO. The additional diagnostic yield of TEE decreased significantly if individuals with known AF or cardiac disease (e.g., congestive heart failure or coronary artery disease) were excluded [25]. Nevertheless, a retrospective analysis identified a non-AF-related major source of embolism in 3.8% of 185 AF patients with acute ischemic stroke undergoing TEE and/or TTE. In addition, 32% of 75 AF patients undergoing TEE had aortic plaques [26].

### Echocardiography vs. magnetic resonance imaging

In a prospective single-center study of 103 ischemic stroke patients, cardiac MRI identified a potential stroke etiology in an additional 6.1% of patients with a cryptogenic stroke according to routine work-up including TEE and in most cases TTE [27]. Four of these five patients had wall motion abnormalities in three or more segments, one was diagnosed with an aortic plaque of ≥4 mm that was missed on echocardiography. However, cardiac MRI failed to reveal a TEE-detected high-risk source of embolism in five cases. In four of these five cases, cardiac MRI was terminated prematurely on behalf of the patient or the urgent need to examine another stroke patient. Of note, late gadolinium enhancement—consistent with previous myocardial infarction—was found in 13 (14.6%) out of 89 stroke patients completing cardiac MRI, of whom only two had known coronary artery disease. The detection of subclinical past myocardial infarction with the help of cardiac MRI in stroke patients is in line with findings from prior studies [28, 29].

Baher et al. [28] evaluated the additional benefit of cardiac MRI in 85 ischemic stroke patients after routine work-up including TTE, but not TEE. Cardiac MRI identified a potential source of embolism in 26% of cases, which were classified as a cryptogenic stroke after routine work-up. Three of these patients had an evident embolic source (LV thrombus [*n* = 2] or complex aortic thrombus). In the remaining three patients, cardiac MRI detected a low-risk embolic source (atrial septal aneurysm [ASA] [*n* = 2] or PFO/ASA).

### Echocardiography vs. computed tomography

Boussel et al. [30] evaluated the role of cardiac CT in a small cohort of patients with cryptogenic stroke. Compared with TEE as reference, CT had a sensitivity of 72% and a specificity of 95% for correct identification of the embolic source. All cases of aortic atheroma diagnosed with TEE were also detected by using CT. Sipola et al. [29] investigated whether combined examination of the heart, aorta, and brain-supplying arteries with CT could improve the diagnosis of stroke etiology compared with standard diagnostics including TTE and TEE in 140 patients with suspected cardioembolic stroke or TIA mainly based on brain imaging findings. The authors conclude that the combined use of CT and TTE/TEE was more sensitive than TTE/TEE alone for detecting at least one high-risk finding. This was mainly due to the detection of previous myocardial infarctions, which were considered as a high-risk embolic source in this study. Computed tomography further identified one additional LV thrombus, but CT was not suitable for diagnosing small LA thrombi. In an earlier prospective single-center study of 137 stroke patients with a high cardiovascular risk profile, cardiac CT was of similar diagnostic value to TEE regarding the identification of high-risk sources of embolism [31]. However, CT failed to detect many low-risk sources of embolism such as PFO/ASA. Furthermore, CT did not yield any additional findings to TEE.

## Cardiac imaging for specific sources of cardioembolism

As indicated earlier, all four imaging modalities differ significantly in terms of their diagnostic performance regarding specific sources of cardioembolism (Table 2).

### Left ventricular thrombus and cardiomyopathy

The development of an LV thrombus is a severe complication of myocardial infarction. Its incidence has decreased significantly with the rise of modern reperfusion measures, but LV thrombus may still occur in up to 8% of patients after ST-elevation myocardial infarction [32, 33]. Left ventricular thrombi are associated with an increased risk for ischemic stroke, which can be reduced by the use of OAC [34, 35].

Cardiac MRI has been shown to have higher sensitivity and specificity for the detection of LV thrombi when compared with TTE and TEE and is considered the gold standard in this setting [36–38]. Nevertheless, TTE is most frequently used for the detection of LV thrombus and may serve as an initial screening test [32]. The diagnostic accuracy of TTE can be improved by the application of endocardial border definition contrast agent resulting in a sensitivity of 61% compared with cardiac MRI [38]. Since many LV thrombi are located in the region of the LV apex, TEE is not superior to TTE for the detection of LV thrombi [23, 36]. If there is high suspicion for an LV thrombus despite a normal finding on TTE, cardiac MRI should be performed for further clarification [39]. If cardiac MRI is not available, CT might also be an option [29, 30, 40, 41].

While LV thrombi are most commonly seen in patients with ischemic cardiomyopathy, other cardiomyopathies are also associated with an increased risk of ischemic stroke. Dilated cardiomyopathy is characterized by a reduction in LV ejection fraction and dilatation of the ventricle, and therefore initial screening can be done by TTE. For further refinement of the etiology, cardiac MRI can be helpful [42]. Reports on the incidence of thromboembolism in patients with dilated cardiomyopathy showed varying results with incidence ranging from 1.7 to 3.5 events per 100 patient-years [43–45]. Another rare cardiomyopathy that may cause cardioembolic stroke is LV non-compaction [46, 47]. It is characterized by multiple prominent ventricular trabeculations with intertrabecular spaces communicating within the ventricular cavity [46]. Transthoracic echocardiography is the initial imaging modality of choice, and application of contrast agent should be considered as it enhances the endocardial border definition unmasking the deep intertrabecular recesses [20, 47]. Again, cardiac MRI might be performed to confirm the diagnosis and exclude LV thrombi.

### Left atrial (appendage) thrombus

The LA and the LAA are the most common sites for intracardiac thrombi and characteristics of both structures have been linked to incident AF and stroke recurrence [48–52]. Left atrial dilation is associated with incident AF and other cardiovascular diseases [50]. Furthermore, the risk of stroke for individuals in sinus rhythm increases along with LA dimensions [53]. In stroke patients with known AF, LA enlargement is a predictor of a higher rate of recurrent stroke or systemic embolism [49]. Before an LAA thrombus manifests, SEC and a reduced LAA flow peak velocity can often be detected [54]. The presence of SEC in patients with AF after stroke has been associated with a poor prognosis and is related to poor long-term functional outcome [48].

Since the sensitivity of TTE for the detection of LA/LAA thrombi is low, TEE is considered the standard diagnostic tool [23]. On the other hand, LA/LAA thrombus is very infrequently detected in the presence of sinus rhythm [22, 55]. Agmon et al. [55] found that patients with LA thrombus and sinus rhythm constitute a high-risk group characterized by structural cardiac abnormalities or previous AF. In a meta-analysis by Romero et al. [56], the mean sensitivity and specificity of cardiac CT was 96% and 92% compared with TEE, respectively. In a further subanalysis of seven mostly prospective, single-center studies in which delayed imaging CT was performed, sensitivity and specificity further increased to 100% and 99%, respectively. In addition to its noninvasive character, CT also offers a superior visualization of the anatomy of the LAA [57]. Although it has not been investigated as extensively as CT, cardiac MRI is also an alternative for the evaluation of the LAA [58]. In a recent retrospective register analysis of patients undergoing pulmonary vein isolation, all thrombi detected by TEE were also found with the use of delayed enhancement cardiac MRI [59]. Ohyama et al. [60] found that in 50 patients with non-valvular AF, all thrombi were successfully identified by cardiac MRI with TEE as the reference standard; but artifacts might result in false-positive findings.

### Patent foramen ovale

A PFO results from the incomplete fusion of the septum primum and the septum secundum in the fossa ovalis [61]. This is a common phenomenon in the general population with a prevalence of about 25% and represents a potential mechanism for paradoxical embolisms via a right-to-left shunt [61, 62]. It is observed significantly more frequently in patients with cryptogenic stroke than in patients with clarified stroke etiology and is associated with an increased risk of stroke recurrence in the presence of an ASA [61, 63]. Recently published controlled trials of patients with cryptogenic stroke showed a significant reduction of stroke recurrence rate by interventional PFO closure compared with standard drug therapy [64, 65]. Based on the currently available data, an interventional PFO closure may be considered in selected patients with cryptogenic stroke, aged ≤60 years, with moderate-to-high atrial shunt volume [66].

The gold standard for the diagnosis of PFO is TEE with a so-called bubble test [67]. An agitated air–saline solution is injected intravenously and a possible bubble transition from the right to the left atrium is examined. If possible, this procedure is repeated while the patient performs a Valsalva maneuver. Screening for a shunt at atrial level can also be performed by TTE with a bubble test, which reliably detects large right-to-left shunts, while smaller shunts are frequently missed [67]. In order to establish the exact anatomy and the precise shunt volume, a TEE examination is necessary. Computed tomography is inferior to TEE with regard to the detection of interatrial septum abnormalities [30, 31, 68]. Kim et al. [68] retrospectively compared the diagnostic performance of TEE and CT in 152 stroke patients. The authors were able to visualize a left-to-right shunt in 21 of the 26 patients in whom a PFO was detected with TEE (sensitivity and specificity of CT were 73% and 98%, respectively). Cardiac MRI currently has no relevance in the diagnosis of a PFO. In a prospective study of patients with cryptogenic stroke, cardiac MRI detected only three out of 31 patients with PFO according to TEE [27]. The superiority of TEE in this regard is in line with data from previous studies, although cardiac MRI performed comparatively better [69, 70].

### Valvular disease

Infective endocarditis (IE) constitutes a severe disease and one of its major complications is septic embolism [71]. Cerebral lesions can be found in up to 80% of patients with left-sided IE, with many episodes being silent and only detectable on cranial imaging [72–74]. The risk of embolism steadily declines after initiation of appropriate antimicrobial therapy [75]. According to current guidelines, TTE is recommended as the first-line imaging modality in cases of suspected IE [71]. However, TEE is superior to TTE in diagnosing IE especially in the case of small vegetations, poor image quality, or prosthetic heart valves [76]. Therefore, if there is persistent clinical suspicion, a TEE examination should be performed despite a normal TTE and repeated if deemed necessary [71]. Furthermore, TEE is obligatory in patients with an abnormal TTE in order to exclude potential complications such as a paravalvular abscess [71]. Besides securing the diagnosis, echocardiography can also aid in the prediction of embolic risk, e.g., vegetation size of >10 mm and mobility are the strongest independent risk factors for embolism [77, 78].

Computed tomography has a good diagnostic accuracy for IE lesions compared with TEE, but small vegetations and leaflet perforations may be missed [79, 80]. On the other hand, CT is often superior to TEE for the assessment of the perivalvular extent of the disease [79, 80]. Furthermore, it can be helpful in cases of prosthetic valve IE, in which acoustic shadows might decrease the sensitivity of TEE [81]. Owing to its lower spatial resolution, MRI does not play a role in the diagnosis of IE.

Besides IE, prosthetic valves might also lead to cerebral embolism as a result of thrombus formation, which is often caused by an inadequate anticoagulation [82]. The incidence rate of mechanical valve thrombosis in patients using anticoagulation is about 0.4 per 100 patient-years, whereas it is believed to be lower in patients with bioprosthesis [83, 84]. The thrombus might cause obstruction of the valve, which can be detected on TTE using the Doppler technique [85]. If suspicion of thrombus formation is high, TEE should be performed for further evaluation. If TEE remains inconclusive, it should be followed by CT for assessment of potential thrombus or pannus [85].

### Cardiac tumors

Primary cardiac tumors are rare and usually benign [86]. Myxomas are the most common primary cardiac tumors and are usually located in the LA. Cerebral embolism occurs in about 30% of cases [87]. Papillary fibroelastomas are by far the most frequent valve-associated tumors and together with myxomas account for the majority of primary cardiac tumors in adults [20, 88, 89]. They are often first diagnosed after thromboembolic stroke [90]. Echocardiography is usually sufficient for the diagnosis of intracardiac tumors with TEE, providing higher sensitivity and spatial resolution [91]. Cardiac CT and MRI generate excellent high-resolution images and can provide important additional information on the extent of the tumor and vascularization. Cardiac MRI is preferred over CT because specific MRI sequences can aid in tissue characterization and in the differential diagnosis including signs of malignancy [89].

## Conclusion

Echocardiography remains the cornerstone of cardiac imaging after acute ischemic stroke. Based on the currently available literature, TTE and TEE should be considered as complementary methods. Transthoracic echocardiography provides comprehensive information on cardiac structure and function and should the first method of choice in most patients. However, TEE might offer additional information especially in patients with a suspected cardioembolic source of stroke and therefore should be considered as the initial examination in these patients. Cardiac CT or MRI may be taken into account as an alternative if TEE is not possible/not available in a timely manner. Currently, the evidence on the effect of cardiac imaging-based changes in clinical management on prognosis after stroke remains scarce, but will hopefully accumulate in the future.

## Abbreviations

AF: Atrial fibrillation
ASA: Atrial septal aneurysm
CT: Computed tomography
ECG: Electrocardiogram
IE: Infective endocarditis
LA: Left atrial/left atrium
LAA: Left atrial appendage
LV: Left ventricular
MRI: Magnetic resonance imaging
OAC: Oral anticoagulation
PFO: Patent foramen ovale
TEE: Transesophageal echocardiography
TIA: Transient ischemic attack
TTE: Transthoracic echocardiography
